# The prevalence and risk of urinary tract infection in malnourished children: a systematic review and meta-analysis

**DOI:** 10.1186/s12887-019-1628-y

**Published:** 2019-07-27

**Authors:** Samuel N. Uwaezuoke, Ikenna K. Ndu, Ikenna C. Eze

**Affiliations:** 1 Department of Pediatrics, College of Medicine, University of Nigeria/University of Nigeria Teaching Hospital, Ituku-Ozalla, Enugu, Postal code: 400001 Nigeria; 2 Department of Pediatrics, Enugu State University Teaching Hospital, Park Lane, Enugu, Nigeria; 3 Swiss Tropical and Public Health Institute, Basel, Switzerland; 4 University of Basel, Basel, Switzerland

**Keywords:** Urinary tract infection, Pooled prevalence, Risk, Malnutrition, Children

## Abstract

**Background:**

There are vast differences in prevalence rates of urinary tract infection (UTI) reported among malnourished children globally. We conducted a systematic review and meta-analysis to provide estimates of pooled prevalence of UTI among these children and combined UTI risk in comparison with their well-nourished counterparts.

**Methods:**

We systematically searched electronic databases (MEDLINE, EMBASE, ISI Web of Science and African Journals Online; date of the last search: 22 December 2018) for studies reporting either the prevalence of UTI in malnourished children or parallel healthy controls. Eligible primary studies were observational studies of children in English Language reporting UTI prevalence with background malnutrition or with enough data to compute these estimates, as well as studies which reported at the same time UTI prevalence in healthy controls. We synthesized published prevalence rates or associations (odds ratios [OR]) between malnutrition and UTI and their 95% confidence intervals (CI) using random effects meta-regression and explored potential heterogeneity determinants using meta-regression analysis. This review is registered with PROSPERO, number- CRD42018084765.

**Results:**

We included 26 cross-sectional and 8 case-control studies reporting on UTI prevalence in malnourished children, and in malnourished children vs. healthy controls, respectively. The pooled prevalence of UTI in 3294 malnourished children was 17% (95% CI, 13, 21%). Heterogeneity was high (I^2^ = 87.6%; Tau^2^ = 0.06) as studies varied in their sample size, degree of malnutrition, and study period. Multivariate meta-regression model, including these factors, explained 34.6% of the between-study variance. Pooled OR of UTI in association with malnutrition in 2051 children (1052 malnourished children vs. 999 controls) was 2.34 (95% CI, 1.15, 3.34), with lower between-study heterogeneity (I^2^ = 53.6%; Tau^2^ = 0.47).

**Conclusions:**

UTI is more prevalent in malnourished children than in their well-nourished counterparts. Screening and treatment for UTI should be incorporated in the management protocol of malnourished children to improve disease outcomes.

**Electronic supplementary material:**

The online version of this article (10.1186/s12887-019-1628-y) contains supplementary material, which is available to authorized users.

## Background

Protein-energy malnutrition (PEM) in children constitutes a global health challenge in developing countries of sub-Saharan Africa and southern Asia [1]. Children with PEM have immunological dysregulation [2] and are thus susceptible to common childhood infections such as infectious diarrhea, pneumonia and bacteremia which, in turn, create a vicious cycle with malnutrition [3]. Similarly, these children are also thought to be mainly predisposed to urinary tract infection (UTI) as the infection risk may also increase with the severity of malnutrition [4], although there appears to be inconsistent evidence linking the degree of malnutrition to higher risk of UTI [5].

The presence of urinary secretory IgA (sIgA) is one of the defense mechanisms against UTI, and its role in UTI episodes has been reported [6–8]. Low urinary sIgA may represent an important predisposing factor to recurrent UTI [9]. Among other effects on the immune system, malnutrition specifically leads to diminished IgA response. A study on experimental animal models showed that dietary protein played a significant and site-specific role in the developmental expression of the secretory immune system, with severe protein malnutrition suppressing this immune arm [10]. Therefore, UTI risk in malnourished children may partly be related to impaired sIgA response.

Several studies have been conducted on UTI prevalence rates and bacterial etiologic patterns in malnourished children across the globe [11–19]. A 2013 systematic review of severely malnourished under-five children revealed a high prevalence of pneumonia (34%), diarrhea (35%) UTI (24%) and bacteremia (17%), with higher mortality rates compared to other children [20]. Furthermore, a more recent non-systematic review revealed vast differences in the prevalence rates with no regional disparities regarding the bacterial isolates, even though sensitivity patterns varied remarkably [5]. There was also no consensus on sex predominance of UTI among malnourished children in some of these studies [11, 12, 18], and controversy still exists on whether UTI risk in these children increases with the severity of malnutrition given the discordant reports about this correlation. Although few studies have compared UTI prevalence in malnourished vs. healthy children, there have been no pooled risk studies directly quantifying the risk of UTI due to malnutrition [21].

We, therefore, conducted a systematic review and meta-analysis to provide estimates of pooled UTI prevalence among malnourished children and of combined UTI risk in comparison with their well-nourished counterparts without age limits and including all degrees of malnutrition. These combined data should provide sufficiently robust evidence to justify the inclusion of screening and treatment of UTI in the management of children with PEM.

## Methods

### Search strategy and selection criteria

We systematically searched electronic databases including MEDLINE, EMBASE, Web of Science, and African Journals Online from inception till 2018 (date of the last search: 22 December 2018). We searched both databases using the following keywords alone and in combination: urinary tract infection, bacteriuria, pyuria, malnutrition, protein-energy malnutrition, severe acute malnutrition, prevalence, incidence, risk, children and infants.

### Inclusion and exclusion criteria

To be included in this review, primary studies had to be observational studies of children (irrespective of origin, ethnic, socioeconomic, and educational background) reporting the prevalence of UTI with background malnutrition or with enough data to compute these estimates. We also included studies which reported an association between malnutrition and UTI or at least UTI prevalence in both malnourished and comparative healthy controls in the same research, enabling the estimation of associations. Both malnutrition and UTI had to be clearly defined in the included studies. Malnutrition had to be defined as a function of weight for age or weight for height using validated reference methods including the World health Organization (WHO)/National Center for Health Statistics (NCHS) [22], Wellcome [23], or Gomez [24] classifications or as mid-arm circumference less than 11 cm. The grade or degree of malnutrition also had to be clearly defined. When absent, we categorized grade I as mild malnutrition, grade II as moderate malnutrition, and grade III as severe malnutrition. UTI had to be defined as significant bacteriuria or pyuria corresponding to the urine sampling method. We included only full-text articles in the English language. We excluded abstracts, letters, reviews, commentaries, editorials, and studies without primary data or explicit description of methods. Two of the investigators (SNU and ICE) independently screened the titles and abstracts of articles retrieved from the literature search. Full texts of articles found potentially eligible were obtained and further assessed for final inclusion. All duplicates were removed during the study selection process. Disagreements were resolved through discussions between the investigators until a consensus was reached.

### Quality assessment

We evaluated the methodological quality of included studies using the Newcastle-Ottawa Scale for assessing non-randomized studies [25]. This scale evaluates case-control and cross-sectional studies using criteria categorized into selection (4 points), comparability (2 points), and exposure/outcome (3 points). Quality Rating was categorized as low (< 7) or high (≥7). Two of the investigators (SNU and ICE) independently assessed study quality, with disagreements resolved by consensus.

### Data extraction

Two of the investigators (SNU and ICE) independently extracted relevant data from individual studies using a preconceived and standardized data-extraction form. Information retrieved included the first author’s name, year of publication, year of study, study setting and country, study design, study population, sample size, and age and sex distribution of participants. We extracted information on urine sampling and analytic methods, UTI and malnutrition diagnostic criteria, the proportion of participants with UTI, and the reported population subgroup differences in proportions. We also extracted information on bacterial isolates and their antibiotic-sensitivity patterns when available. Where relevant data were not available, we contacted the corresponding author to request for the information. We assessed the inter-rater agreement for study inclusion and data extraction using Cohen’s κ coefficient [26].

### Data analysis

#### A meta-analysis of prevalence studies

The synthesized study-specific estimates were pooled using random effects meta-regression model to obtain an overall summary estimate of the prevalence across studies, after stabilizing the variance of individual studies with the use of the Freeman-Tukey double arcsine transformation [27]. Random-effect models give more weight to smaller studies and have wider confidence intervals because they consider potential variation between the actual effects that all included studies estimate, in addition to their within-study variance. We calculated the I^2^ and tau^2^ to assess between-study heterogeneity. We assessed publication bias using funnel plots and the formal Egger [28], and Begg’s tests [29]. We considered any test *p*-values less than 0·05 to be indicative of significant publication bias. We assessed subgroup differences in prevalence estimates based on factors such as sex (males/females), age group (< 18 months/≥18 months), malnutrition severity (moderate or severe /mild or mixed), region of origin (Africa/Others), study design (cross-sectional/ case-control), study quality (low/high), year of study (< 2000/≥2000) and urine-collection method (one method/multiple methods or unspecified). We performed sensitivity analyses, including fixed effect meta-regression, leave-one-out random effects meta-regression to explore the stability of our pooled prevalence estimate. We assessed the sensitivity of the combined estimates to the exclusion of studies with < 30 participants, and studies where urine sampling method, urinalysis method or definition of UTI were not stated. We performed meta-regression analysis using study-level covariates as predictors of study-level estimates to explore the determinants of potential heterogeneity in our pooled estimates, in bivariate and multivariate models.

#### A meta-analysis of association studies

We pooled the reported or derived estimates of association (odds ratios (OR) and 95% CI) between malnutrition and UTI from included case-control studies also using random effects meta-regression model. When no UTI was reported for the control group, we added 0.5 to all four related groups to estimate the OR and 95% CI in the affected studies [30, 31]. We also calculated the I^2^ and tau^2^ to assess between-study heterogeneity and evaluated publication bias using funnel plots and the formal Egger [28], and Begg’s tests [29]. We performed subgroup analysis, including stratification by matching (i.e., if the studies matched the Cases and Controls by at least age or sex, or both). We also performed sensitivity analyses, including fixed-effect and leave-one-out meta-regression. Given the limited number of association studies (*n* = 8), we could not perform further meta-regression analyses. Data were analyzed using STATA version 14.0 for Windows (STATA Corporation, Texas).

For reporting, we adhered to the Preferred Reporting Items for Systematic Reviews and Meta-Analysis [32], and the Meta-analysis Of Observational Studies in Epidemiology guidelines [33]. This systematic review is registered with PROSPERO, number- CRD42018084765.

## Results

### Study selection

**Fig. 1:**
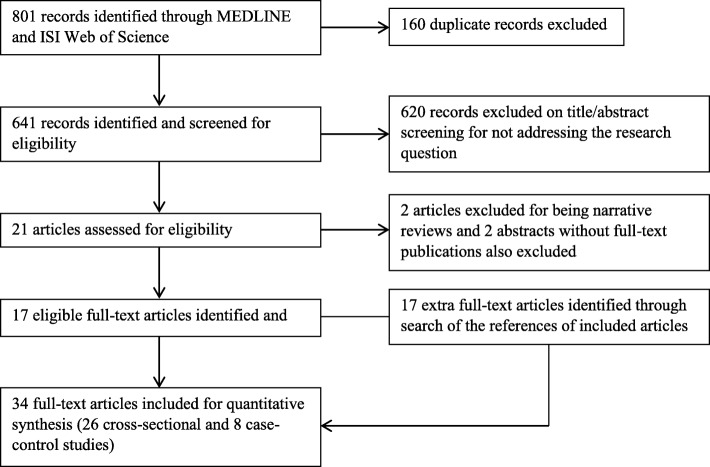
Algorithm for inclusion in the present study

We identified 1478 records following a combined search of MEDLINE, EMBASE, ISI Web of Science, and African Journals Online databases. Exclusion of duplicates and non-pertinent articles yielded 35 articles, of which 33 met the eligibility criteria. A further search of the references of these articles yielded an additional item. Thus, the present review includes 34 full-text articles, either reporting UTI in malnourished children only or parallel with well-nourished children. Details of the article-selection algorithm are presented in Fig. 1.

### Characteristics of included articles

Overall, we included 26 cross-sectional (76%) and 8 case-control studies (24%). All included studies were hospital-based studies. Most of the studies were from African countries including South Africa [13–15, 34–36] Nigeria [11, 37–40], Uganda [41, 42], Kenya [17, 43] Tanzania [18, 44], Ethiopia [16] Niger [12], Sudan [45], and Gambia [19]. Other studies were conducted in Turkey [46, 47], India [4, 48, 49], Pakistan [50, 51], Bangladesh [52], Thailand [53], Iran [54], Australia [55], Peru [56], and Jamaica [57]. Sample size varied, with 18% of the studies having < 50 participants. Eight cross-sectional studies (31%) primarily investigated UTI in malnourished children [13, 14, 17, 18, 35, 46, 53, 57], whereas the remaining 18 studies (69%) reported UTI as a secondary outcome in the broader context of bacterial infections in malnourished children [11, 12, 15, 16, 19, 37–45, 50–52, 55]. Most of the case-control studies (88%) primarily investigated UTI occurrence in malnourished children vs. healthy controls [4, 34, 47–49, 54, 56], whereas one (12%) reported UTI prevalence in both groups in the broader context of bacterial infections in children [36]. The pooled study population included 3294 malnourished children from 26 cross-sectional studies and 2051 children (1052 malnourished and 999 controls) from the 8 case-control studies, for estimating the pooled prevalence of UTI and pooled OR and 95%CI of UTI with malnutrition, respectively (Table 1).

**Table 1:** Characteristics of studies on malnutrition and urinary tract infection

Source	Country of study	Study setting and period	Study population	Study design
Philips I et al. 1968 [41]	Uganda	Infantile Malnutrition Research Unit, Medical Research Unit, Kampala. Study period not specified	75 malnourished children admitted consecutively over a nine-month period. Age range not specified	Cross-sectional
Brooke O. G et al. 1973 [57]	Jamaica	Tropical Metabolism Research Unit, University of West Indies. Study period not specified	95 malnourished children (39 females; 56 males) admitted over an 18-month period, aged 4–35 months (mean 12.6 months)	Cross-sectional
Buchanan N et al. 1973 [34]	South Africa	Baragwanath Hospital, Johannesburg. Study period not specified	30 admitted malnourished children aged 7–36 months (mean of 15 months)	Cross-sectional
Morehead D et al. 1974 [53]	Thailand	Anemia and Malnutrition Research Centre, Chang Mai Hospital, Chang Mai between June 1969 and April 1970	35 consecutively admitted malnourished children (18 females; 17 males) aged 10–50 months (mean of 22 months)	Cross-sectional
Brown KH et al. 1981 [52]	Bangladesh	Children’s Nutrition Unit, Dacca, between January 1976 and April 1976	100 admitted (50 males and 50 females) children aged 18–30 months (median 20 months)	Cross-sectional
Morton RE et al. 1982 [40]	Nigeria	Pediatric out-patient department of Ahmadu Bello University Teaching Hospital, Zaria. Study period not specified	65 malnourished children visiting the outpatient clinic over a six-month period, aged 0–120 months	Cross-sectional
Berkowitz FE 1983 [15]	South Africa	General Pediatric wards of Baragwanath hospital, Johannesburg between December 1981 and November 1982	16 admitted malnourished children (part of 68; 35 males and 33 females) aged 4–48 months (mean 16.9 months)	Cross-sectional
Oyedeji G 1989 [39]	Nigeria	Children’s ward, Wesley Guild Hospital Ilesha, between January 1985 and December 1986	73 admitted malnourished children (30 females; 43 males) aged 12–96 months (mean 22.6 months)	Cross-sectional
Isaack H et al. 1992 [44]	Tanzania	Pediatric wards of Muhimbili Hospital Dar es Salaam. Study period not specified	164 admitted malnourished children (89 males, 75 females) aged 2–59 months (mean 19 months) who had not been on any antibiotics in the previous 24 h, and studied over a two-week period.	Prospective; Cross-sectional.
Kala UK et al. 1992 [13]	South Africa	Baragwanath Hospital, Johannesburg. Study period not specified.	75 consecutively-admitted malnourished children (44 males, 31 females) aged 3–60 months (mean 15.4 months)	Cross-sectional
Ighogboja et al. 1993 [38]	Nigeria	Children’s ward, Jos University Teaching Hospital between January 1991 and December 1991	130 admitted malnourished children (52 females; 78 males) aged 11–96 months (mean 22.8 months)	Cross-sectional
Shimeles D et al. 1994 [16]	Ethiopia	Ethio-Swedish Children’s Hospital, Addis Ababa, between January 1 and December 31, 1992	19 children (part of 90 admitted malnourished children, 40 males, and 50 females) Aged 4–60 months (median 15 months)	Cross-sectional
Reed P et al. 1995 [14]	South Africa	Shongwe Mission Hospital, Shongwe, Malelane between September 1992 and April 1993	134 presenting malnourished children (73 males, 61 females) aged 1–59 months (median 17 months) not using antibiotics in the previous 24 h	Prospective; Cross-sectional
Ekanem EE et al. 1997 [37]	Nigeria	University Teaching Hospital Calabar. Study period not specified	27 children (part of 37 admitted malnourished children aged 3–60 months (mean 22 months) recruited for a case-control study on differences in CRP and C3 levels in protein-energy malnutrition with and without infection)	Cross-sectional
Caksen H et al. 2000 [46]	Turkey	Department of Pediatrics, Yüzüncü Yil University, between May 1998 and November 1998	103 admitted malnourished children aged 1.6–30 months (mean 11.6 months)	Cross-sectional
Rabasa AI et al. 2002 [11]	Nigeria	Pediatric wards of University of Maiduguri Teaching Hospital between January 1994 and December 1996	194 admitted malnourished children (128 males and 66 females) aged 3–60 months (mean 17.6 months)	Cross-sectional
Russell B et al. 2004 [55]	Australia	Alice Springs Hospital, Alice Springs between January 2000 and September 2001	55 admitted malnourished Central Australian Indigenous children aged 0.6-41 months (mean of 8.6 months) sampled from medical records	Retrospective cross-sectional
Noorani N et al. 2005 [43]	Kenya	Pediatric Filter Clinic of Kenyatta National Hospital, Nairobi between March 2003 and October 2003	91 consecutively presenting malnourished children (45 males, 46 females) aged 2–60 months (mean 18 months)	Cross-sectional
Bachou H et al. 2006 [42]	Uganda	Pediatric wards of Mulago Hospital, Kampala between September–November 2003 and September–December 2004	315 consecutively admitted malnourished children (196 males,119 females) with a median age of 17 months	Cross-sectional
Okomo UA et al. 2011 [19]	The Gambia	Pediatric ward, Medical Research Council Hospital, Fajara, between November 2007 and December 2008	97 children (part of 140 admitted malnourished children aged 6–59 months (median 19.1 months) without non-nutritional causes of edema, chronic infection or antibiotic use in the previous two weeks	Prospective; Cross-sectional
Suliman OSM et al. 2011 [45]	Sudan	Pediatric wards of the Khartoum Teaching Hospital and Soba University Teaching Hospital between December 1992 and May 1993	49 admitted malnourished children aged 6–60 months (mean 22 months)	Cross-sectional
Page A et al. 2013 [12]	Niger	intensive therapeutic feeding center in the Maradi region between November 2007 and July 2008	300 (out of a total of 311 admitted malnourished children (170 males and 141 females) aged 6–59 months (median 13 months)	Cross-sectional
Sameen I and Moorani N 2014 [50]	Pakistan	Nutritional Rehabilitation Unit, National Institute of Child Health, Karachi between January 2012 and June 2012	130 admitted malnourished children (78 males and 52 females) aged 1–59 months (mean: 16.8 months)	Cross-sectional
Ahmed M et al. 2015 [18]	Tanzania	Pediatric wards of Bugando Medical Centre, Mwanza between September 2012 and January 2013	402 admitted malnourished children (173 males and 229 females) aged 6–60 months (median 17 months)	Cross-sectional
Anjum M et al. 2016 [51]	Pakistan	Nutritional Rehabilitation Unit of National Institute of Child Health, Karachi between October 2014 and March 2015	78 admitted malnourished children (39 males and 39 females) aged 2–60 months (mean 18 months)	Cross-sectional
Thuo N et al. 2017	Kenya	pediatric ward at the Centre for Geographical Medicine Research, Coast between June 2005 and June 2007	498 admitted malnourished children (271 males, 227 females) with a median age of 22.4 months	Prospective; Cross-sectional
Buchanan N et al. 1971 [34]	South Africa	Baragwanath Hospital, Johannesburg. Study period not specified	125 outpatient children [75 malnourished (5 females; 70 males) and 50 controls (5 females; 45 males)] without urinary tract signs or symptoms, studied over two months. The age range of malnourished children was 8–96 months (mean 43 months), and the age range of controls was 2–108 months (mean 30 months)	Case-control
Freyre EA et al. 1973 [56]	Peru	Department of Pediatrics, Universidad Nacional de San Agustin, Arequipa. Study period not specified	200 malnourished children (108 females and 92 males) and 118 controls (61 females and 57 males) outpatients and admitted patients, aged 3–36 months (mean 20 months)	Case-control
Bodaghi E et al.1978 [54]	Iran	Children’s Hospital Medical Center, Tehran. Study period not specified	667 outpatient or admitted children (348 malnourished [143 females; 205 males] and 319 controls [140 females; 174 males] aged 2–24 months and not having any antimicrobial therapy in the past 48 h.	Prospective; Case-control
Banarpurmath C et al. 1994	India	Pediatric wards and Out-patient Department, Chigateri General Hospital, Devangere, between April 1989 and April 1990	141children [88 admitted malnourished children selected from the Pediatric wards and 53 out-patient controls aged 12–60 months	Case-control
Jeena PM et al. 1995 [36]	South Africa	King Edward VIII Hospital, Durban in November 1992	32 malnourished children and 148 controls aged 0–144 months	Case-control
Caksen H et al. 2001 [47]	Turkey	Department of Pediatrics, Yüzüncü Yil University, Study period not specified	146 admitted malnourished children (69 females; 77 males) [47 malnourished and 99 controls] without symptoms suggesting urinary tract infection, aged 0.9–15 months (mean 4.6 months)	Case-control
Bagga A et al. 2003 [4]	India	All India Institute of Medical Sciences, New Delhi between September 1997 and July 1998	224 consecutive out-patient children (112 malnourished [47 females; 65 males] and 112 controls [47 females; 65 males]) aged 6–60 months (mean of 35.5 months)	Case-control
Gopal G and Premalatha R 2014 [49]	India	Department of Pediatrics, Mysore Medical College and Research Institute, Mysore between November 2008 and August 2010	250 children (150 admitted malnourished (93 males and 57 females) and 100 outpatient controls (55 males and 45 females)) aged 6–60 months (mean 27 months)	Case-control

Most of the studies included participants who had moderate-to-severe malnutrition (76%), while the rest had mixed malnourished populations (24%). There were differences in urine-sampling methods with most studies employing two or multiple methods (74%) including combinations of suprapubic aspiration, mid-stream urine or urine bags [4, 16, 18, 34–36, 38–40, 48, 49, 52, 53, 57], compared to a single method (26%) in their study population [11–13, 17, 43, 47, 54, 56] (Table 2). There was uniformity in the definition of UTI across studies, which was consistently applied to the urine-sampling method (Table 2). Although all studies examined the prevalence of UTI in malnourished children, 38% of the included studies did not explicitly describe urine-collection method, urine analytic method, or UTI (Table 2).

**Table 2:** Definition of malnutrition and urinary tract infections across included studies

Source	Definition of malnutrition	Degree of malnutrition	Urine sampling method	Urinalysis method	Definition of UTI
Cross-sectional studies					
Philips I et al. 1968 [41]	Marasmus or kwashiorkor	Severe malnutrition, including kwashiorkor (84%) and marasmus (16%).	urine bag or suprapubic aspiration (if specimen using bag is contaminated)	Culture (details not specified)	Not specified
Brooke O. G et al. 1973 [57]	Protein-energy malnutrition	Severe malnutrition (100%)	Sterile urine bags or suprapubic tap	microscopy and culture (details not specified)	> 10000 organisms/ml of urine confirmed by suprapubic tap (if a bad specimen was initially used)
Buchanan N et al. 1973 [35]	Kwashiorkor, marasmic kwashiorkor, marasmus or underweight for age (below the third percentile)	Severe malnutrition including Kwashiorkor: 46.7%), marasmic Kwashiorkor (23.3%), Marasmus (20%) and Underweight for age (10%)	Midstream urine or sterile urine bags	Uricult dip-slide (nutrient agar on one side and McConkey’s agar on the other side, with 13 cm^2^ areas for each medium) incubated at 37 °C for 16–24 h.	> 10^5^ organisms/ml of urine
Morehead D et al. 1974 [53]	Kwashiorkor, marasmus or marasmic kwashiorkor	Moderate/severe malnutrition including marasmus: (22.9%) marasmic kwashiorkor (51.4%) and Kwashiorkor (25.7%).	Suprapubic tap or urethral catheterization	Plating on sheep blood and McConkey agar within one hour of the collection (or immediately put in a refrigerator to be plated within 24 h) and Gram staining of urine samples	> 10^5^ organisms/ml of urine
Brown KH et al. 1981 [52]	Marasmus, marasmic kwashiorkor or kwashiorkor	Severe malnutrition including marasmus (57.1%), marasmic kwashiorkor (28.6%) and kwashiorkor (14.3%).	Suprapubic aspiration or freshly voided specimen	Microscopy and culture (details not specified)	≥10^5^ colonies/ml of urine (mid-stream) or ≥ 1 organism (suprapubic aspiration)
Morton RE et al. 1982 [40]	Kwashiorkor or marasmus	Severe malnutrition, including kwashiorkor (52.3%) and marasmus (47.7%).	Suprapubic aspiration and mid-stream urine.	Culture using McConkey and blood agar for 18 h	Any growth from suprapubic aspiration or ≥ 10^5^ from mid-stream urine
Berkowitz FE 1983 [15]	Marasmus, marasmic kwashiorkor and kwashiorkor	Severe malnutrition including kwashiorkor (68%), marasmus (12%) and marasmic Kwashiorkor (20%).	Suprapubic aspiration	Microscopy and culture (details not specified).	≥One organism/ml of urine
Oyedeji G 1989 [39]	Marasmus, kwashiorkor or marasmic kwashiorkor, plus at least one feature of the disease compelling hospitalization (severe dermatoses with extensive wet areas, severe edema, intractable diarrhea, intolerance of oral fluids and feeds, hypothermia and severe mental apathy)	Severe malnutrition, including kwashiorkor (67.1%) and marasmic Kwashiorkor (32.9%).	mid-stream urine or suprapubic tap	microscopy, culture (details not specified) and sensitivity	Not specified
Isaack H et al. 1992 [44]	Marasmus, marasmic kwashiorkor or kwashiorkor	Severe malnutrition including marasmus (55.5%), kwashiorkor (23.8%) and marasmic kwashiorkor (20.7%).	Not specified	Urine culture using Mc Conkey’s and blood agar	Not specified
Kala UK et al. 1992 [13]	Underweight, marasmus, kwashiorkor or marasmic kwashiorkor	All forms of malnutrition including underweight (29.3%), marasmus (13.3%), kwashiorkor (41.3%) and marasmic kwashiorkor (16%)	Suprapubic aspiration	Dip-slide cultures (Uricult®-Boehringer Mannheim and incubated at 37 degrees C for 24-48 h) and microscopy	Presence of any growth on dip-slide culture
Ighogboja et al. 1993 [38]	Marasmus, kwashiorkor or Marasmic-kwashiorkor	Severe malnutrition, including marasmic kwashiorkor (46.2%), kwashiorkor (29.2%) and marasmus (24.6%).	Mid-stream urine or suprapubic tap	Microscopy, culture (medium not specified) and sensitivity	Not specified
Shimeles D et al. 1994 [16]	Marasmus, Marasmic-Kwashiorkor or Kwashiorkor	Severe malnutrition, including marasmus (48.9%), kwashiorkor (18.9%) and marasmus (32.2%).	Suprapubic aspiration or sterile bags	Microscopy and culture (details not specified)	Not specified
Reed P et al. 1995 [14]	Nutritional dwarfism, kwashiorkor, marasmus or Marasmic Kwashiorkor	All including kwashiorkor (53.7%), nutritional dwarfism (32.8%), marasmus (8.2%) and marasmic kwashiorkor (5.2%).	Suprapubic aspiration	Dipstick urinalysis for leukocytes and nitrites using Combur-9 strips (Boehringer Mannheim); Culture in cystine lactose electrolyte deficient medium, blood agar and McConkey agar (Bio Lab media, Merck Ltd., Johannesburg) incubated at 37 degrees overnight; Antimicrobial Sensitivity using Kirby-Bauer disk-diffusion method.	Any growth from suprapubic aspiration
Ekanem EE et al. 1997 [37]	Kwashiorkor, marasmus or marasmic-kwashiorkor	Severe malnutrition including kwashiorkor (51.9%), marasmus (25.9%) and marasmic-kwashiorkor (22.2%)	Not specified	Urine culture (medium not specified)	Not specified
Caksen H et al. 2000 [46]	Weight for age below the 90th percentile (Grade I: 76–90% or Grade II: 61–75% or Grade III: < 60%)	Mild to severe malnutrition (100%)	Not specified	microscopy and culture (details not specified)	≥10^5^ colonies/ml of urine with the same organism
Rabasa AI et al. 2002 [11]	Marasmus, marasmic kwashiorkor and kwashiorkor	Severe malnutrition including marasmus (67%), marasmic kwashiorkor (13.4%) and kwashiorkor (19.6%).	Suprapubic aspiration	Culture on McConkey’s agar, cysteine lactose electrolyte deficient medium and incubated 18-24 h at 37.1 °C and sensitivity using the disc method	≥One organism/ml of urine.
Russell B et al. 2004 [55]	Weight loss resulting in downwards crossing of two major percentile lines or no weight gain (weight and weight for age z-score)	Mild/ moderate/ severe (76% gained no weight in the past 2–3 months, and 24% crossed down two major percentile lines)	Not specified	Not specified	Not specified
Noorani N et al. 2005 [43]	Kwashiorkor, marasmus or marasmic-kwashiorkor	Severe malnutrition including kwashiorkor (22%), marasmus (66%), and marasmic-kwashiorkor (12%)	Suprapubic aspiration	Urine culture using CLED medium incubated overnight and sensitivity using diffusion technique	Not specified
Bachou H et al. 2006 [42]	Presence of edema and/or weight for height z score > −3 of the NCHS/WHO reference values	Severe malnutrition including severe wasting (45.4%) and edematous malnutrition (54.6%)	Not specified	Urine culture and sensitivity	Not specified
Okomo UA et al. 2011 [19]	Very low weight for height (below −3z scores of the median NCHS/WHO growth standards, visible severe wasting, or the presence of nutritional edema.	Severe acute malnutrition (32.1% had edema with or without weight for height below -3SD)	Suprapubic aspiration (if < 12 months) or urethral catheterization or clean-catch sample (children > 12 months or in cases of dry suprapubic tap).	Microscopy, culture (cysteine lactose electrolyte deficient (CLED) agar incubated overnight at 37 degrees) and sensitivity	≥10^5^ colonies/ml of urine (mid-stream) or ≥ 1 organism/ml of urine (suprapubic aspiration)
Suliman OSM et al. 2011 [45]	Kwashiorkor, marasmus or marasmic-kwashiorkor	Severe malnutrition including marasmus (46.9%), marasmic-kwashiorkor (34.7%) and kwashiorkor (18.4%)	Not specified	Urine microscopy, culture, and sensitivity	≥Five pus cells/HPF and/or positive cultures
Page A et al. 2013 [13]	Weight-for-height < 3z scores of the median WHO growth standards and/or mid-arm circumference, 110 mm and/or bipedal edema. Complicated malnutrition if accompanied by anorexia and/or Kwashiorkor with bilateral pitting edema and/or another severe condition.	Severe malnutrition with 15.4% being edematous	Foley catheter	Dipstick urinalysis, Culture using CHRO Magar inoculation plate and sensitivity using Kirby Bayer disk diffusion method on Mueller-Hinton agar.	Single pathogen ≥10^4^/ml (*Escherichia coli*) or ≥ 10^5^/ml (others) regardless of the number of leukocytes in urine OR bacteriuria ≥10^3^/ml (*Escherichia coli*) or 10^4^/ml (others in the presence of at least 10^4^ leukocytes/ml in the urine
Sameen I and Moorani N 2014 [50]	Weight for height Z scores (below -3 SD with or without bilateral pitting edema and any of the following: anorexia, severe anemia, high fever, severe dehydration, and systemic infection.	Severe malnutrition including severe wasting (80.8%) and edematous malnutrition (19.2%)	Not specified	Urine culture and sensitivity	Not specified
Ahmed M et al. 2015 [18]	Weight-for-height < 3 SD of the z score according to WHO Classification (Mild (−1 SD) or moderate (−2 SD) or severe (−3SD)	All. Mild (36.6%), moderate (19.2%) and severe (44.3%)	Mid-stream urine (if > 24 months) or suprapubic aspiration (if < 24 months)	Culture (cysteine lactose electrolyte deficient agar (CLED), MacConkey and blood agar plates (Oxoid UK) incubated at 37 degrees for 24 h. Drug susceptibility using disc diffusion method	Any growth from suprapubic aspiration or ≥ 10^5^/ml of mid-stream urine
Anjum M et al. 2016 [51]	Presence of bilateral pitting edema or weight for height z score > − 3 of the NCHS/WHO reference values	Severe malnutrition including marasmus (82%) and edematous malnutrition (18%)	Not specified	Microscopy and culture	Not specified
Thuo N et al. 2017	Pedal edema (kwashiorkor or marasmic kwashiorkor) or weight for height Z score ≤ − 3 or mid-arm circumference < 11 cm (if length > 65 cm)	Severe (36% with edema)	Mid-stream urine	Microscopy, culture in cystine lactose electrolyte deficient agar at 37 degrees and sensitivity	Growth of a single pathogen at ≥50 colony forming units/μl
Case-control studies					
Buchanan N et al. 1971 [34]	Atrophic malnutrition or kwashiorkor	Moderate/severe malnutrition including kwashiorkor: (33.3%) and atrophic malnutrition: (66.7%)	midstream urine or sterile urine bags or suprapubic aspiration	Uricult dip-slide (nutrient agar on one side and McConkey’s agar on the other side, each medium covering 13 cm^2^ slide area) incubated at 37 °C for 16–24 h. Confirmation using conventional pour plate method for suprapubic urine specimen	> 10^5^ organisms/ml of urine
Freyre EA et al. 1973 [56]	Marasmus, Marasmic Kwashiorkor or Kwashiorkor	Severe malnutrition including marasmus (39%), kwashiorkor (20%) and marasmic kwashiorkor (41%).	Sterile plastic urine collector	Culture using the Henrich method	≥10^5^ colonies/ml of urine
Bodaghi E et al.1978 [54]	Less than 75% standard weight for age (Grade I: 70–75%; Grade II: 60–70%; Grade III: 50–60%; Grade IV: 40–50%)	Mild /moderate /severe including Grade I (20%), Grade II (20%), Grade III (33%) and Grade IV (27%)	Sterile urine bags	Culture on blood, nutrient and eosin methylene blue agar media	2–3 consecutive specimen revealing 10^5^ colonies/ml of urine with the same organism of the same serotype
Banarpurmath C et al. 1994	Weight for age of < 60% (Grade I: 71–80% or Grade II: 61–70% or Grade III: 51–60% or Grade IV: < 50%)	Severe malnutrition including Grade III (70.5%) and Grade IV (29.5%).	Suprapubic aspiration (children < 3 years old) and mid-stream urine (> 3 years)	Direct microscopic examination, gram stain, and culture (details not specified)	≥10^5^ colonies/ml of urine (mid-stream) or ≥ 1 organism (suprapubic aspiration)
Jeena PM et al. 1995 [36]	Protein-energy malnutrition defined according to conventional clinical features	All (groups not specified)	Urethral catheterization, suprapubic aspiration or clean-catch sample	microscopy, culture, and sensitivity	≥10^5^ colonies/ml of urine (mid-stream or clean catch) or > 10^3^ organisms (suprapubic aspiration);
Caksen H et al. 2001 [47]	Weight for age below the 90th percentile (Grade I: 76–90% or Grade II: 61–75% or Grade III: < 60%)	Mild to severe malnutrition (combined Grade I-III: 31%)	Sterile urine bags	Urine microscopy and culture (sheep agar and eosin methylene blue agar plates incubated at 35 °C for 24–48 h)	Two consecutive specimens revealing 10^5^ colonies/ml of urine with the same organism
Bagga A et al. 2003 [4]	< 80% weight for age (Grade I: 71–80% or Grade II: 61–70% or Grade III: 51–60% or Grade IV: < 50%)	Moderate/ severe, including Grade II (49.1%), Grade III (45.5%) and Grade IV (5.4%).	Suprapubic tap or clean-catch	Microscopy (WBC counting using a Neubauer counting chamber; and Gram staining) and culture	Any growth on urine specimen collected by suprapubic aspiration or > 10^5^ organisms/ml of clean-catch urine
Gopal G and Premalatha R 2014 [49]	Less than 70% of the expected weight for age (Grade II: 60–69.9% or Grade III: 50–59.9% or Grade IV: < 50%)	Moderate to severe malnutrition including Grade II (34%), Grade III (37%) or Grade IV (29%) malnutrition	Suprapubic aspiration (if < 36 months) and clean catch (if ≥36 months)	Urine microscopy and culture (culture medium not specified)	> 5 pus cells/high power field or a positive urine culture

*UTI* urinary tract infection, *NCHS* National Center for Health Statistics, *WHO* World Health Organization

### UTI prevalence in malnourished children

**Fig. 2:**
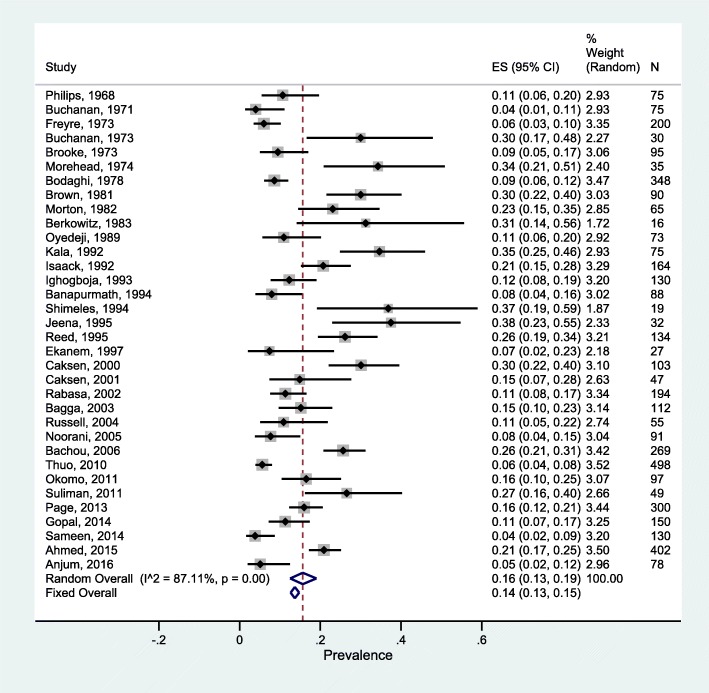
Overall UTI prevalence in malnourished children

**Fig. 3:**
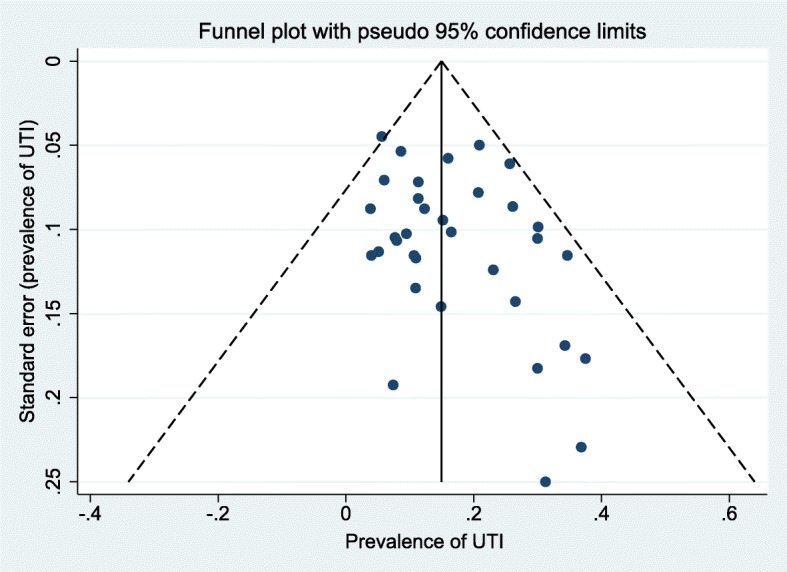
Funnel plot for visualization of publication bias with studies reporting UTI prevalence

As shown in Fig. 2, the pooled random-effects prevalence of UTI in 3294 malnourished children was 17% (95% CI: 13, 21%). Heterogeneity was high across studies (I^2^ = 87.6%; *P* < 0.001; Tau^2^ = 0.06). Subgroup analyses showed significant differences by degree of malnutrition (severe: 15% (95% CI: 11, 19%); mild/mixed: 25% (95% CI: 19, 32%); P_heterogeneity_: 0.01) and sample size (Sample size < 50: 27% (95% CI: 18, 36%); Sample size ≥50: 16% (95% CI: 12, 20%); P_heterogeneity_: 0.02), and borderline-significant differences by year of study (year < 2000: 21% (95% CI:16,26%); year≥2000: 14% (95% CI: 9, 19%); P_heterogeneity_: 0.06). We did not observe significant differences by age group (P_heterogeneity_: 0.21), study region (P_heterogeneity_: 0.68) and study quality (P_heterogeneity_: 0.33). Although the difference by urine sampling method was non-significant (P_heterogeneity_: 0.29), the prevalence of UTI in studies which applied suprapubic aspiration or sterile catheterization alone was 14% (95% CI: 7, 22%) while that of those combining different methods was 18% (95% CI: 14, 23%). Sex-specific prevalence of UTI in malnourished children was similar among the six studies reporting these estimates (UTI prevalence in males: 23% (95% CI: 14, 32%); females: 20% (95% CI: 14, 27%); P_heterogeneity_ = 0.61) (Table 3). Figure 3 shows the funnel plot for visualization of publication bias. We observed minimal evidence for publication bias as both Egger’s (*P* = 0.15) and Begg’s tests (*P* = 0.35) were non-significant. Further sensitivity analyses revealed the robustness of our findings. Fixed-effects prevalence of UTI in malnourished children was 15% (95% CI: 14, 17%) (Fig. 2) whereas exclusion of studies with <30 participants or not specifying urine sampling or analytic method or UTI definition yielded a random-effects pooled prevalence of 17% (95% CI: 13, 21%) and 20% (95% CI, 14, 27%) respectively (Additional file 1: Table S2).

**Table 3:** Subgroup random-effects prevalence estimates of urinary tract infection in malnourished children

Variable	Subgroup	N	Prevalence	Within-group heterogeneity estimates			Between-group heterogeneity estimates	
			% (95% CI)	Q-statistic	*P*-value	I^2^ (%)	Q-statistic	*P*-value
Sex	Males	6	23% (14, 32%)	30	< 0.001	83.2	0.3	0.61
	Females	6	20% (14, 27%)	14	0.01	64.8		
Age	< 18 months	13	18% (13, 23%)	109	< 0.001	87.8	0.2	0.21
	≥18 months	13	16% (10, 23%)	74	< 0.001	87.3		
Year of publication	< 2000	14	21% (16, 26%)	52	< 0.001	74.9	1.6	0.06
	≥2000	12	14% (9, 19%)	129	< 0.001	91		
Malnutrition severity	Severe	20	15% (11, 19%)	129	< 0.001	91.4	3.6	0.01
	Mixed	6	25% (19, 32%)	17	< 0.001	70.4		
Region	Africa	19	21% (17, 25%)	137	< 0.001	86.9	0.2	0.68
	Others	7	16% (7, 27%)	64	< 0.001	90.6		
Study quality	Low	23	17% (12, 22%)	171	< 0.001	87.2	0.9	0.33
	High	3	21% (16, 26%)	*n.a.*	*n.a.*	*n.a.*		
Sample size	< 50	6	27% (18, 36%)	9	0.11	44.2	5.8	0.02
	≥50	20	16% (12, 20%)	178	< 0.001	89.3		
Urine sampling	One method^*^	6	14% (7, 22%)	53	< 0.001	90.5	1.1	0.29
	Multiple methods/ not specified	20	18% (14, 23%)	112	< 0.001	83.1		

All estimates were derived from meta-analytic models with Freeman-Tukey double arcsine transformation. *n.a.* not applicable due to very low sample size in the group. *One method includes either suprapubic aspiration or sterile catheterization

Results from meta-regression analyses including study-level covariates showed the degree of malnutrition, sample size and year of study to be significant predictors of prevalence rates, explaining 24.1, 5.8 and 5.7% of the between-study variance respectively in the bivariate models, respectively. Degree of malnutrition remained significant in the multivariate meta-regression model that also included sample size and year of study. Studies, including severely-malnourished children, reported a lower prevalence of UTI compared to a milder/mixed group (OR: 0.90 (95% CI: 0.83, 0.97)). Although statistically non-significant, prevalence of UTI also decreased with sample size (OR: 0.92 (95% CI: 0.83, 1.02)) and studies published from 2000 (OR: 0.95 (95% CI: 0.89, 1.02)). This multivariate meta-regression model explained 33.9% of the between-study variance in the pooled estimates (Table 4).

**Table 4:** Meta-regression estimates to explain the prevalence of urinary tract infection in malnourished children

Variable	Subgroup	Bivariate model	Adjusted R^2^ (%)	Multivariable model	Adjusted R^2^ (%)
		OR (95% CI)		OR (95% CI)	
Malnutrition severity	Mild or mixed	Ref.	24.06	Ref.	34.63
	Moderate/Severe	0.90 (0.83, 0.99)**		0.90 (0.83, 0.97)**	
Sample size	< 50	Ref.	5.77	Ref.	
	≥50	0.91 (0.82, 1.01)*		0.92 (0.83, 1.02)	
Year of publication	< 2000	Ref.	5.79	Ref.	
	≥2000	0.94 (0.87, 1.01)*		0.95 (0.89, 1.02)	
Age	< 18 months	Ref.	2.17	–	
	≥18 months	0.95 (0.88, 1.03)		–	
Urine sampling	Multiple/unspecified method	Ref.	−0.53	–	
	One method	0.96 (0.87, 1.05)		–	
Study quality	Low	Ref.	−1.76	–	
	High	1.04 (0.92, 1.17)		–	
Region	Others	Ref.	−3.77	–	
	Africa	1.02 (0.93, 1.12)		–	

Regression estimates were derived from linear regression models with urinary tract infection prevalence as an outcome. All models included the 26 studies reporting the prevalence of UTI in malnourished children. *P < 0.1; **P ≤ 0.05

### Risk of UTI in malnourished children vs. healthy controls

**Fig. 4:**
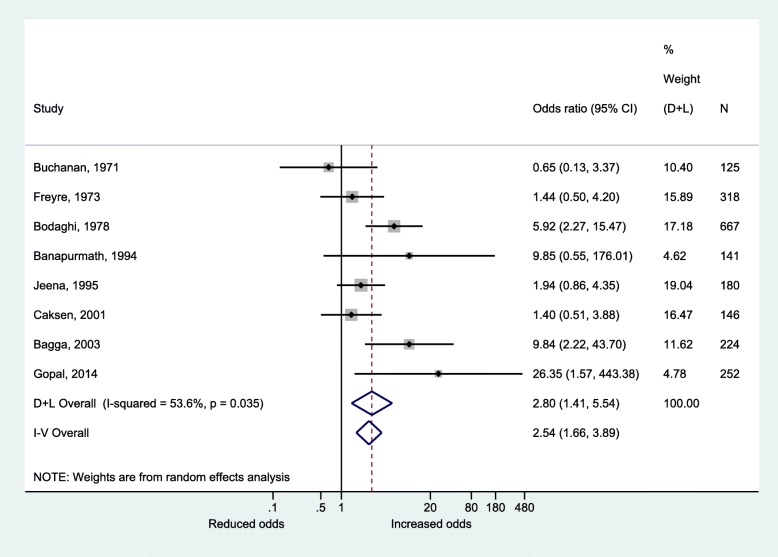
Meta-analysis of overall UTI prevalence rate

**Fig. 5:**
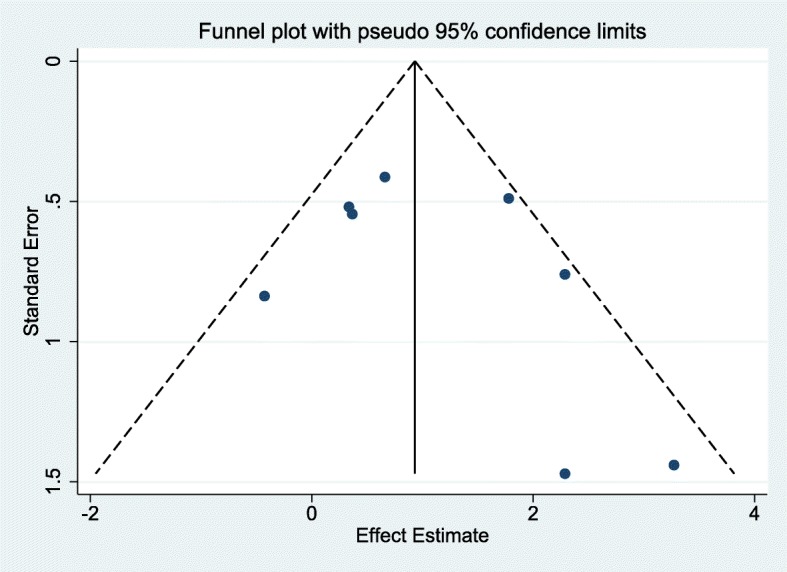
Funnel plot for visualization of publication bias with the case-control studies

Random-effects pooled OR of UTI in 1052 malnourished children, and 999 controls were 2.80 (95% CI: 1.41, 5.54). We observed moderate heterogeneity in across studies (I^2^ = 53.6%; *P* = 0.04; Tau^2^ = 0.47) (Fig. 4). Stratifying by matching criterion showed differences in random effects associations between UTI and malnutrition (OR matched studies: 5.67 (1.39, 23.2); I^2^ = 56.7%; *P* = 0.07; Tau^2^ = 1.09; OR in unmatched studies: 2.04 (0.91, 4.57); I^2^ = 57.4%; *P* = 0.07; Tau^2^ = 0.38). Figure 5 shows the funnel plot for visual assessment of publication bias within the case-control studies. We also observed minimal evidence for publication bias given the non-significant Egger’s (*P* = 0.34) and Begg’s tests (*P* = 0.90). Sensitivity analyses revealed robust effect estimates. Fixed effect pooled OR of UTI was 2.50 (95% CI: 1.66, 3.89) (Fig. 3). Leave-one-out random effects OR of UTI ranged from 2.34 (1.19, 4.62) to 3.26 (1.63, 6.50). We observed the smallest heterogeneity (I^2^ = 47.2%; *P* = 0.08; Tau^2^ = 0.41) on the exclusion of the study by Banapurmath and Jayamony [48].

### Bacterial isolates and antibiotic-sensitivity patterns

Urine culture was performed by 28 (82%) of the included studies. Of the 27 studies that reported urinary bacterial isolates, *Escherichia coli* was the predominant isolate in 25 (93%) of them, whereas *Klebsiella spp.* was predominant in 2 (7%). Most common bacterial strains included gram negative coliforms, including *Escherichia coli* (100%), *Klebsiella spp.* (81%), *Proteus spp.* (41%), *Pseudomonas spp.* (33%), *Enterobacter spp.* (22%), and *Citrobacter spp.* (15%). Other reported gram-negative bacterial isolates include *Salmonella spp.* (7%), *Serratia spp.* (7%), *Hafnia alvei* (4%) and *Morganella morganii* (4%). Gram-positive isolates were less prevalent and included *Staphylococcus spp.* (7%), *Enterococcus spp.* (7%), and *Streptococcus faecalis* (4%) as well as the fungus, *Candida albicans* (4%). Antibiotic sensitivity tests were performed by 13 (38%) studies, with different sensitivity patterns (Table 5).

**Table 5:** Prevalence of urinary tract infections (UTI) and bacterial isolates in malnourished children across included studies

Source	Prevalence of UTI	Subgroup differences	Bacterial Isolates	Antibiotic sensitivity
Cross-sectional studies				
Philips I et al. 1968 [41]	10.7%	Not specified	*Escherichia coli* (75%); *Proteus species* (12.5%); *Klebsiella spp* (12.5%).	Not done
Brooke O. G et al. 1973 [57]	9.5%	Males:12.5%; Females: 5.1%	*Escherichia coli* (44.5%); *Klebsiella spp* (44.5%); *Proteus spp* (11%)	Not done
Buchanan N et al. 1973 [35]	30%	Not specified	*Escherichia coli* (55.6%); *Klebsiella spp* (22.2%); *Proteus mirabilis* (22.2%)	Not done
Morehead D et al. 1974 [53]	34.3%	Not specified	*Escherichia coli* (58.3%); *Enterobacter spp* (25%); *Proteus mirabilis* (16.7%); *Proteus spp* (8.3%); *Klebsiella spp* (8.3%); *Staphylococcus aureus* (8.3%); Microaerophilic streptococci (8.3%); *Streptococcus fecalis* (8.3%); Non-hemolytic streptococci (8.3%)	*Escherichia coli*; cephalothin (8%), ampicillin (4%), tetracycline (18%), kanamycin (30%), colistin (75%), gentamicin (68%) and chloramphenicol (14%). *Klebsiella spp*, *Proteus spp* and *Enterobacter spp* also had low sensitivity to all antibiotics. *Staphylococcus aureus*; Cephalothin (100%), kanamycin (90%) and gentamicin (98%), but less sensitive to the other antibiotics
Brown KH et al. 1981 [52]	30%	Males: 24%; Females: 36%	*Escherichia coli* (96%); *Pseudomonas spp* (4%)	Not done
Morton RE et al. 1982 [40]	23%	Not specified	*Escherichia coli* (48%); *Klebsiella spp* (39%); *Citrobacter spp* (5%).	Not done
Berkowitz FE 1983 [15]	31%	Not specified	*Escherichia coli* (100%)	Not done
Oyedeji G 1989 [39]	11%	Not specified	*Escherichia coli* (25%); *Klebsiella spp* (75%)	Not done
Isaack H et al. 1992 [44]	21%		*Escherichia coli* (52.9%); *Klebsiella spp* (41.2%); *Pseudomonas spp* (2.9%); Other coliforms (2.9%).	*Escherichia coli* and *Klebsiella spp*; Gentamycin (100%), cotrimoxazole (15, 14%), nitrofurantoin (26, 22%); *Klebsiella spp*; Chloramphenicol (100%). *Escherichia coli*; Chloramphenicol (8%) and penicillin (0%).
Kala UK et al. 1992 [13]	35%	Males: 47.7%; Females: 16.1%. Underweight: 31.8%; Marasmus: 10%; Kwashiorkor: 41.9%; and Marasmic Kwashiorkor: 41.7%.	*Escherichia coli* (84.6%); *Proteus mirabilis* (7.7%); *Klebsiella pneumoniae* (3.8%); *Pseudomonas aeruginosa* (3.8%).	Not done
Ighogboja et al. 1993 [38]	12.3%	Not specified	*Escherichia coli* (37.5%); *Klebsiella spp* (37.5%); *Pseudomonas spp* (18.8%); *Candida albicans* (6.2%)	Sensitive to gentamicin, cefuroxime axetil, ceftazidime and ofloxacin
Shimeles D et al. 1994 [16]	37%	Not specified	*Escherichia coli* (42.9%); *Klebsiella pneumoniae* (42.9%) (3/7); *Citrobacter spp* (14.3%)	Not done
Reed P et al. 1995 [14]	26%	Males: 30.1%; Females: 21.3%. Nutritional dwarfism: 29.5%; Marasmus: 18.2%; Kwashiorkor: 23.6%; and Marasmic kwashiorkor: 42.9%.	*Escherichia coli* (42.9%); *Enterobacter spp* (14.3%); *Klebsiella spp* (14.3%); *Citrobacter spp* (8.6%); *Hafnia alvei* (2.8%); *Proteus mirabilis* (2.8%); *Pseudomonas spp* (2.8%); *Serratia spp* (2.8%); *Salmonella typhi* (2.8%). *S aureus* (2.8%); *Enterococcus faecalis* (2.8%)	*Escherichia coli*; Nalidixic acid (100%), nitrofurantoin (92.3%), cephradine (84.6%), gentamicin (84.6%), cotrimoxazole (0%) and amoxicillin (7.7%). *Enterobacter spp*; Gentamicin (100%), cephradine (100%), nalidixic acid (100%), nitrofurantoin (60%), cotrimoxazole (40%) and amoxicillin (0%). *Klebsiella spp*; Nitrofurantoin (100%), nalidixic acid (100%), cephradine (80%), gentamicin (80%), cotrimoxazole (0%) and amoxicillin (20%). *Citrobacter spp*; Gentamicin (100%), cephradine (100%), nalidixic acid (100%) and nitrofurantoin (100%), amoxicillin (0%), cotrimoxazole (0%). Other gram negatives; Gentamicin (100%), cephradine (100%) and nalidixic acid (100%). *Staphylococcus aureus*; Amoxicillin (100%), gentamicin (100%), cephradine (100%), nitrofurantoin (100%) cotrimoxazole (0%) and nalidixic acid (0%). *Enterococcus faecalis*; Amoxicillin (100%), cotrimoxazole (100%), nalidixic acid (100%), nitrofurantoin (100%), gentamicin (0%) and cephradine (0%).
Ekanem EE et al. 1997 [37]	7.4%	Not specified	*Klebsiella spp* (50%); *Pseudomonas spp* (50%).	Not done
Caksen H et al. 2000 [46]	30.1%	No significant difference between UTI and degree of malnutrition	*Escherichia coli* (54.8%): *Klebsiella pneumoniae* (9.6%); *Proteus mirabilis* (9.6%); *Enterobacter cloacae* (6.4%); *Klebsiella oxitoca* (6.4%); *Morganella morganii* (3.2%); *Citrobacter freundii* (3.2%); *Enterobacter aerogenes* (3.2%); *Salmonella spp* (3.2%)	All isolates sensitive to gentamicin (100%). *Escherichia coli and Klebsiella spp;* Cotrimoxazole (18 and 20%), ceftriaxone (82 and 100%), cefotaxime (82 and 100%) and ciprofloxacin (82 and 100%) respectively.
Rabasa AI et al. 2002 [11]	11.35%	Kwashiorkor:10.5%; Marasmus: 10.1%; Marasmic kwashiorkor: 15.3%	*E. coli* (45.4%); *Klebsiella spp* (27.3%); *Pseudomonas spp* (13.6%); *Staphylococcus aureus* (13.6%)	95% of Gram negatives were sensitive to gentamycin and/or ofloxacin; *Staphylococcus aureus* sensitive to gentamycin, co-trimoxazole, ceftazidime, and clavulanic acid potentiated amoxicillin (Augmentin®). All gram negatives showed poor sensitivity to co-trimoxazole and nitrofurantoin.
Russell B et al. 2004 [55]	11%	Not specified	Not specified	Not done
Noorani N et al. 2005 [43]	7.6%	Not specified	*Klebsiella spp* (57%); *E. coli* (43%).	*Klebsiella spp* and *Escherichia coli*; Amikacin (100%), ceftriaxone (100%), ciprofloxacin (100%), ampicillin (0%), ceftazidime (83.3%), cefuroxime (83.3%; 50%), chloramphenicol (0%; 66.7%), cotrimoxazole (16.7%; 0%) and gentamicin (66.7%; 83.3%).
Bachou H et al. 2006 [42]	25.7%	HIV-positive: 30%; HIV- negative: 23%	Not done	Not done
Okomo UA et al. 2011 [19]	16.5%		*Escherichia coli* (58.8%); *Klebsiella spp* (17.6%); *Enterobacter cloacae* (5.9%); *Proteus spp* (5.9%); *Providencia alkali* (5.9%); *Pseudomonas aeruginosa* (5.9%).	*Escherichia coli*; Gentamicin (100%), ciprofloxacin (100%), cefuroxime (100%), cefotaxime (100%), nitrofurantoin (100%), chloramphenicol (77%), ampicillin (0%) and cotrimoxazole (0%).
Suliman OSM et al. 2011 [45]	28.5%	Not specified	Not done	Not done
Page A et al. 2013 [12]	16%	Males: 12.2%; Females: 20.6%. Age < 12 months: 24%; Age > 12 months: 10.9%. Fever: 16.7%; No fever: 15.9%.	*Escherichia coli*: 77%; *Klebsiella pneumoniae*: 14.6%; *Proteus mirabilis*: 4.2%; *Proteus penneri*: 2.1%; *Enterococcus faecium*: 2.1%	*Escherichia coli*; amoxicillin (0%), cotrimoxazole (5%), amoxicillin-clavulanic acid (39%), cephalothin (56%), cefoxitine (95%) cefotaxime (95%), ceftazidime (95%), imipenem (100%), gentamicin (90%) amikacin (100%) nalidixic acid (88%), ofloxacin (90%) and Extended Spectrum Beta-Lactamase (ESBL; 95%); *Klebsiella spp*; Amoxicillin (0%), cotrimoxazole (33%) amoxicillin-clavulanic acid (42%), cephalothin (58%), cefoxitine (92%) cefotaxime (92%), ceftazidime (92%), imipenem (100%), gentamicin (58%) amikacin (100%) nalidixic acid (100%), ofloxacin (100%) ESBL (92%)
Sameen I and Moorani N 2014 [50]	3.8%	Not specified	Not done	Not done
Ahmed M et al. 2015 [18]	20.65%	Males: 19.6%; Females: 21.4%. Fever: 22.6%; No fever: 18.4%. Mild malnutrition: 14.3%; Moderate malnutrition: 18.2%; Severe malnutrition: 27%. HIV-positive: 19.35%; HIV-negative: 20.75%.	*Escherichia coli*: 41.2%; *Klebsiella pneumoniae*: 23.8%; Other gram negatives (*Proteus spp, Enterobacter spp, Citrobacter spp, Serratia spp*): 34.5%	*Escherichia coli*; Ampicillin (3%) gentamicin (57%), ciprofloxacin (86%), amoxicillin/clavulanic acid (14%), ceftriaxone (66%), ceftazidime (60%) and etrapenem (97%). *Klebsiella pneumoniae*; Ampicillin (0%), Gentamicin (30%), ciprofloxacin (85%), amoxicillin/clavulanic acid (15%), ceftriaxone (50%), ceftazidime (40%) and etrapenem (100%); Others; Ampicillin (0%), gentamicin (34%), ciprofloxacin (97%), ceftriaxone (48%), ceftazidime (48%) and etrapenem (100%).
Anjum M et al. 2016 [51]	5%	Not specified	Not done	Not done.
Thuo N et al. 2017	6%	Not specified	Coliforms (100%)	Cotrimoxazole (7%), gentamycin (57%), nalidixic acid (86%) and nitrofurantoin (79%).
Case-control studies				
Buchanan N et al. 1971 [34]	4%	Not specified	*Escherichia coli* (67%); *Proteus spp* (33%)	Not done
Freyre EA et al. 1973 [56]	6%	Males: 4.3%; Females: 7.4%. No significant differences with the severity of clinical malnutrition.	*Escherichia coli* (76.5%). Others not reported.	Not done
Bodaghi E et al.1978 [54]	8.6%	Males:8.8%; Females: 8.4%	*Escherichia coli* (90%); *Klebsiella spp* (3%); *Proteus spp* (9%)	Not done
Banapurmath C et al. 1994 [48]	8.3%	Not specified	*Escherichia coli* (42.9%); *Klebsiella spp* (14.3%); *Proteus spp* (28.6%); *Enterobacter spp* (14.3%)	Not done
Jeena PM et al. 1995 [36]	37.5%	Not specified	Total gram negatives (79%); *Escherichia coli* (53%),	All gram-negatives; Nalidixic acid (100%), amikacin (100%), cephalexin (91%) and Augmentin® (94%), cotrimoxazole (58%), trimethoprim (69%) and ampicillin (86%)
Caksen H et al. 2001 [47]	14.8%	Not specified	*Escherichia coli* (27.7%); *Klebsiella pneumoniae* (61.1%); *Staphylococcus aureus* (5.6%); *Enterobacter spp* (5.6%)	Not done
Bagga A et al. 2003 [4]	15.2%	Moderate malnutrition: 7.3%; Severe malnutrition: 22.8%. Diarrhea: 23.3%; No diarrhea: 10.1%	*Escherichia coli* (64.7%); *Klebsiella spp* (23.5%); *Proteus spp* (5.9%); *P aeruginosa* (5.9%)	Most organisms sensitive to co-trimoxazole, amoxicillin, cephalexin, ciprofloxacin, gentamicin and ceftriaxone
Gopal G and Premalatha R 2014 [49]	11.3%	Males: 10.8%; Females: 12.2%. Grade II: 11.8%; Grade III: 16.4% and Grade IV malnutrition: 4.5%	Not done	Not done

### Comorbidities of UTI in malnourished children

The most commonly reported morbidities in malnourished children were diarrhea or gastroenteritis (53%; *n* = 18) [12, 15, 16, 37–39, 41, 42, 44–46, 48, 50, 52–56], respiratory diseases (including pneumonia, tuberculosis, respiratory tract infection and abnormal chest radiographs; 47%; *n* = 16) [12, 16, 19, 37–39, 41, 42, 45, 46, 48, 50, 52–55] and bacteremia or sepsis (47%; *n* = 16) [12, 14–17, 19, 37–39, 41–44, 50, 52, 53]. Six studies reported co-occurrence of UTI with at least one of these common malnutrition-associated morbidities [12, 14, 19, 39, 48, 54]. Only 27% (*n* = 7) of the cross-sectional studies on UTI in malnourished patients investigated renal urinary tract malformations in their UTI patients [4, 36, 48, 49, 54, 56], reporting a combined malformation prevalence of 14% in these patients. In contrast, 75% (*n* = 6) of the case-control studies utilized radiological investigations to identify malformations as a risk factor for UTI in their patients, reporting a prevalence of 34% (*n* = 80) among the malnourished children and a prevalence of 4% (*n* = 4) among the healthy controls.

## Discussion

This paper is the first PROSPERO-registered systematic review on UTI among malnourished children. In this review and meta-analysis of data from 34 studies involving 3294 malnourished children, we found a pooled UTI prevalence of 17% and pooled OR of 2.34 for UTI in association with malnutrition in 2051 children (1052 malnourished children versus 999 controls). Our combined prevalence rate is at variance with the rate of 24.1% reported in a systematic review on the justification for antibiotic use in children with uncomplicated severe acute malnutrition (SAM) [20]. The disparity could be due to differences in the number of reviewed studies (26 in the current study versus 10 in the comparative study), and may also be explained by the predominant age bracket of the malnourished children reviewed by these authors [20], which fell within the period of pre-toilet/toilet training: a phase that contributes to UTI risk in childhood [21]. The systematic review by Alcoba et al. specifically selected studies that investigated the prevalence of other infections, such as human immunodeficiency virus, bacteremia, lower respiratory tract infection, and diarrhea in strictly SAM and not-only-SAM children [20]. However, the prevalence rate from our review is similar to the 11–16.5% prevalence reported in the selected studies from the West African sub-region [11, 12, 19, 38, 39], India [4, 49], Turkey [47], and Australia [55].

We found no significant sex predominance in the few studies that reported a sex-specific prevalence of UTI in malnutrition. This finding is inconsistent with the known epidemiologic trajectory of UTI in which prevalence rates for both sexes may be the same during infancy, but show male predominance in the neonatal period and female preponderance during early childhood and the period of toilet training [5]. More importantly, the later female dominance may be due to anatomical differences where the proximity of the urethral opening to the vagina may facilitate urethral contamination [58]. In addition, recent evidence suggests that the sex differences in the reticuloendothelial system which provides innate immunity against microbes may also contribute to the sex differences in UTI prevalence rates [59]. Thus, irrespective of nutritional status, female sex remains a risk factor for UTI in childhood. We also noted that UTI risk was increased by the severity of malnutrition. Its prevalence was slightly higher in children aged less than 18 months. Although the latter observation may be related to exposure to gut uropathogenic bacterial flora during the period of pre-toilet training, the former agrees with the report of one of the selected studies which showed a direct correlation of UTI risk with the severity of malnutrition [4]. It is however in contrast with the findings of studies in Nigeria [11], and South Africa [14], which did not establish any significant change in UTI prevalence rates for the different grades of malnutrition. The lower prevalence of UTI in the severely malnourished children may be related to their lower efficiency in immune response due to lack of immune cells and immune dysfunction which characterize severe malnutrition [60]. Although non-significant, the higher prevalence of UTI in studies combining several sampling techniques (that included less sterile methods) might have been due to contamination in the collection process. But the allowance of up to 10^5^ colonies per ml in the diagnosis of UTI (when the reference method of suprapubic aspiration is not used) limits outcome misclassification, and could explain the non-significant difference observed in our study.

Our finding of a positive and significant pooled risk of UTI in malnourished children compared to healthy control is not surprising given their higher susceptibility to infections based on their immune dysregulation. We also found a consistent report of higher occurrence of other infections across studies which investigated other concurrent infections. Malnourished children also had higher prevalence of urinary tract anomalies, which is a known risk factor for UTI [61].

Another key finding in our systematic review is the predominance of *Escherichia coli* and other gram-negative coliforms as the bacterial isolates. This trend is similar in both malnourished and non-malnourished children. It is trite to mention that exposure of children to infection with gut uropathogens (during pre-toilet and toilet-training periods) is a putative UTI risk factor, which may partly explain this observation. Apart from the role of malnutrition in causing diminished IgA response (including sIgA), the reduced transferrin levels in malnourished children may result in the circulation of free unbound iron, which creates a favorable environment for the growth of gram-negative bacteria leading to gram-negative sepsis and subsequently UTI via the hematogenous route [5].

There are substantial differences in the antibiotic-sensitivity patterns of the predominantly isolated gram-negative bacteria, including *Escherichia coli*. Our observation across the reviewed studies clearly shows no defined pattern of sensitivity and resistance to the tested antibiotics. This finding underscores the need for a periodic institution-based update of antibiotic-sensitivity trends. Relying on previous sensitivity reports as guides for empirical therapy may result in poor outcomes for new cases of UTI in malnourished children.

The strengths of our study include its broad approach in identifying relevant articles, and the consideration of both UTI prevalence in malnourished children, and the risk of UTI in malnourished children vs. controls. We explored publication bias, and the determinants of high heterogeneity observed in our estimates. Our inclusion of a large number of studies also allowed for sensitivity analyses, which confirmed the robustness of our pooled estimates. However, our research has some limitations. First, we observed high heterogeneity across the studies included in the combined prevalence estimates. While we identified some factors that explained some of the between-study heterogeneity, other unmeasured factors could have also contributed as we could only explain 34.6% of this heterogeneity. The inclusion of earlier studies may have biased our pooled estimates given the continuous updates of definitions and management protocols for childhood diseases. However, the definition of UTI and other methodologies were quite similar across included studies, and although stratification by year of publication showed higher prevalence of UTI among older studies (before 2000; pooled prevalence of 21%), the prevalence of newer studies (2000 and later; pooled prevalence of 14%) was similar to the overall pooled random effects estimate (17%). Year of publication was also not a significant determinant of between-study heterogeneity in the meta-regression model (Table 4). Our observation of the absence of publication bias in the pooled OR of UTI might have been due to the small number of studies, as there is a high risk of non-detection of publication bias in meta-analyses that include less than ten publications [62].

In conclusion, our systematic review has shown that UTI is more prevalent in malnourished children than in their well-nourished counterparts. It has been suggested that if children at high risk of UTI like those with malnutrition were screened, the number of children missed or treated inappropriately could be reduced [63]. We recommend the incorporation of screening and treatment for UTI into the management protocol for malnourished children to improve disease outcomes.

## Additional file

Additional file 1: **Table S1.** Study quality scores based on the Newcastle-Ottawa scale for non-randomized studies. **Table S2.** Sensitivity analyses of UTI prevalence in malnourished children and association of UTI and malnutrition in malnourished children and healthy controls. **Table S3.** Leave-one-out sensitivity analyses of random-effects prevalence of urinary tract infection in malnourished children. **Table S4.** Leave-one-out sensitivity analyses of random-effects association between malnutrition and urinary tract infection in children. (DOCX 28 kb)

## Data Availability

All data generated or analyzed during this study are included in this published article (and its supplementary information files).

## Abbreviations

CI: Confidence interval
OR: Odds ratio
PEM: Protein-energy malnutrition
SAM: Severe acute malnutrition
sIgA: Secretory immunoglobulin A
UTI: Urinary tract infection
