# Supplementary material for: The prevalence and risk of urinary tract infection in malnourished children: a systematic review and meta-analysis
Source: BMC Pediatr. 2019 Jul 27;19:261. doi: 10.1186/s12887-019-1628-y (PMC6660684; doi:10.1186/s12887-019-1628-y)
Supplement: Supplementary file 1 — Table S1. Study quality scores based on the Newcastle-Ottawa scale for non-randomized studies. Table S2. Sensitivity analyses of UTI prevalence in malnourished children and association of UTI and malnutrition in malnourished children and healthy controls. Table S3. Leave-one-out sensitivity analyses of random-effects prevalence of urinary tract infection in malnourished children. Table S4. Leave-one-out sensitivity analyses of random-effects association between malnutrition and urinary tract infection in children. (DOCX 28 kb) [file 12887_2019_1628_MOESM1_ESM.docx]

SUPPLEMENTARY MATERIAL

**Urinary tract infection in children with malnutrition: a systematic review and meta-analysis**

- Samuel N Uwaezuoke.^1^ ([snuwaezuoke@yahoo.com](mailto:snuwaezuoke@yahoo.com))
- Ikenna K Ndu.^2^ ([ikennandu@gmail.com](mailto:ikennandu@gmail.com))
- Ikenna C Eze.^3, 4^ ([Ikenna.eze@swisstph.ch](mailto:Ikenna.eze@swisstph.ch))

1. Department of Paediatrics, University of Nigeria Teaching Hospital, Ituku-Ozalla, Enugu
2. Department of Paediatrics, Enugu State University Teaching Hospital, Park Lane, Enugu
3. Swiss Tropical and Public Health Institute, Basel, Switzerland
4. University of Basel, Basel, Switzerland

Supplementary Table 1: Study quality scores based on the Newcastle-Ottawa scale for non-randomized studies

| Source | Selection | Comparability | Outcome/Exposure | Total |
| --- | --- | --- | --- | --- |
| Philips I et al. 1968 | 3 | 0 | 3 | 6 |
| Buchanan N et al. 1971 | 4 | 0 | 2 | 6 |
| Brooke O.G et al. 1973 | 3 | 0 | 3 | 6 |
| Buchanan N et al. 1973 | 3 | 0 | 3 | 6 |
| Freyre EA et al. 1973 | 4 | 1 | 3 | 8 |
| Morehead D et al. 1974 | 3 | 0 | 3 | 6 |
| Bodaghi E et al.1978 | 4 | 0 | 3 | 7 |
| Brown KH et al. 1981 | 3 | 0 | 3 | 6 |
| Morton RE et al. 1982 | 2 | 0 | 3 | 5 |
| Berkowitz FE 1983 | 2 | 0 | 3 | 5 |
| Oyedeji G 1989 | 3 | 0 | 3 | 6 |
| Isaack H et al. 1992 | 3 | 0 | 3 | 6 |
| Kala UK et al. 1992 | 3 | 0 | 3 | 6 |
| Ighogboja et al. 1993 | 3 | 0 | 3 | 6 |
| Banapurmath C et al. 1994 | 4 | 1 | 2 | 7 |
| Shimeles D et al. 1994 | 3 | 0 | 3 | 6 |
| Jeena PM et al. 1995 | 4 | 0 | 2 | 6 |
| Reed P et al. 1995 | 3 | 0 | 3 | 6 |
| Ekanem EE et al. 1997 | 2 | 0 | 3 | 5 |
| Caksen H et al. 2000 | 3 | 0 | 3 | 6 |
| Caksen H et al. 2001 | 4 | 0 | 2 | 6 |
| Rabasa AI et al. 2002 | 3 | 0 | 3 | 6 |
| Bagga A et al. 2003 | 4 | 2 | 3 | 9 |
| Russell B et al. 2004 | 3 | 0 | 3 | 6 |
| Noorani N et al. 2005 | 3 | 0 | 3 | 6 |
| Bachou H et al. 2006 | 4 | 0 | 3 | 7 |
| Thuo N et al. 2010 | 3 | 0 | 3 | 6 |
| Okomo UA et al. 2011 | 3 | 0 | 3 | 6 |
| Suliman OSM et al. 2011 | 3 | 0 | 3 | 6 |
| Page A et al. 2013 | 4 | 0 | 3 | 7 |
| Gopal G and Premalatha R 2014 | 4 | 1 | 2 | 7 |
| Sameen I and Moorani N 2014 | 3 | 0 | 3 | 6 |
| Ahmed M et al. 2015 | 4 | 1 | 3 | 8 |
| Anjum M et al. 2016 | 3 | 0 | 3 | 6 |

Supplementary Table 2: Sensitivity analyses of UTI prevalence in malnourished children and association of UTI and malnutrition in malnourished children and healthy controls.

| Model | N | Pooled estimate (95% CI) | Q-statistic for heterogeneity | P-value (Q) | Proportion of variation attributable to heterogeneity (I^2^; %) | Between-study variance (Tau^2^) |
| --- | --- | --- | --- | --- | --- | --- |
| Random-effects prevalence model | 34 | 16% (13%, 19%) | 255.5 | <0.001 | 87.1% | 0.05 |
| Fixed effects prevalence model | 34 | 14% (13%, 15%) | - | - | 0 | - |
| Random-effects prevalence model excluding studies with <30 participants | 31 | 15% (12%, 19%) | 241.9 | <0.001 | 87.6 | 0.05 |
| Random-effects prevalence model excluding studies where urine collection or urinalysis method or urinary tract infection definition was not specified | 21 | 16% (12%, 21%) | 175 | <0.001 | 88.3 | 0.05 |
| Random-effects association model | 8 | 2.80 (1.41, 5.54)** | 15.1 | 0.04 | 53.6 | 0.47 |
| Fixed-effects association model | 8 | 2.50 (1.66, 3.89)** | 15.1 | 0.04 | 53.6 | 0.47 |
| Random-effects association model in matched studies | 4 | 5.67 (1.39, 23.2)** | 6.9 | 0.07 | 56.7 | 1.09 |
| Random-effects association model in unmatched studies | 4 | 2.04 (0.91, 4.57)* | 7.1 | 0.07 | 57.4 | 0.38 |
| Fixed-effects association model in matched studies | 4 | 3.65 (1.65, 8.11)** | 6.9 | 0.07 | 56.7 | - |
| Fixed-effects association model in unmatched studies | 4 | 2.20 (1.33, 3.64)** | 7.1 | 0.07 | 57.4 | - |

*P<0.1; **P>0.05

Supplementary Table 3: Leave-one-out sensitivity analyses of random-effects prevalence of urinary tract infection in malnourished children

| Study omitted | Prevalence of urinary tract infection (95% CI) | Q-statistic for heterogeneity | P-value (Q) | Proportion of variation attributable to heterogeneity (I^2^; %) | Between-study variance (Tau^2^) |
| --- | --- | --- | --- | --- | --- |
| Philips, 1968 | 16% (13%, 19%) | 255.3 | <0.001 | 87.5 | 0.06 |
| Buchanan, 1971 | 16% (13%, 20%) | 246.9 | <0.001 | 87 | 0.05 |
| Brooke, 1973 | 16% (13%, 19%) | 254.2 | <0.001 | 87.4 | 0.06 |
| Buchanan, 1973 | 15% (12%, 19%) | 251 | <0.001 | 87.3 | 0.05 |
| Freyre, 1973 | 16% (13%, 20%) | 240.6 | <0.001 | 86.7 | 0.05 |
| Morehead, 1974 | 15% (12%, 19%) | 247.5 | <0.001 | 87.1 | 0.05 |
| Bodaghi, 1978 | 16% (13%, 19%) | 244.6 | <0.001 | 86.9 | 0.06 |
| Brown, 1981 | 15% (12%, 19%) | 241.8 | <0.001 | 86.8 | 0.05 |
| Morton, 1982 | 15% (12%, 19%) | 252.2 | <0.001 | 87.3 | 0.05 |
| Berkowitz, 1983 | 15% (12%, 19%) | 252.8 | <0.001 | 87.3 | 0.05 |
| Oyedeji, 1989 | 16% (13%, 19%) | 255.4 | <0.001 | 87.5 | 0.06 |
| Isaack, 1992 | 15% (12%, 19%) | 256 | <0.001 | 87.2 | 0.06 |
| Kala, 1992 | 15% (12%, 18%) | 237.5 | <0.001 | 86.5 | 0.05 |
| Ighogboja, 1993 | 16% (13%, 19%) | 255.6 | <0.001 | 87.5 | 0.06 |
| Banapurmath, 1994 | 16% (13%, 19%) | 252.9 | <0.001 | 87.3 | 0.05 |
| Shimeles, 1994 | 15% (12%, 19%) | 251 | <0.001 | 87.2 | 0.05 |
| Jeena, 1995 | 15% (12%, 19%) | 245.9 | <0.001 | 86.9 | 0.05 |
| Reed, 1995 | 15% (12%, 19%) | 243.2 | <0.001 | 86.8 | 0.05 |
| Ekanem, 1997 | 16% (13%, 19%) | 255.1 | <0.001 | 87.5 | 0.05 |
| Caksen, 2000 | 15% (12%, 19%) | 239.7 | <0.001 | 86.7 | 0.05 |
| Caksen, 2001 | 16% (13%, 19%) | 255.9 | <0.001 | 87.5 | 0.05 |
| Rabasa, 2002 | 16% (13%, 19%) | 254.6 | <0.001 | 87.4 | 0.06 |
| Bagga, 2003 | 16% (12%, 19%) | 255.8 | <0.001 | 87.5 | 0.06 |
| Russell, 2004 | 16% (13%, 19%) | 255.6 | <0.001 | 87.5 | 0.05 |
| Noorani, 2005 | 16% (13%, 19%) | 252.4 | <0.001 | 87.3 | 0.05 |
| Bachou, 2006 | 15% (12%, 19%) | 231.6 | <0.001 | 86.2 | 0.05 |
| Okomo, 2011 | 16% (12%, 19%) | 255.4 | <0.001 | 87.5 | 0.06 |
| Suliman, 2011 | 15% (12%, 19%) | 258 | <0.001 | 87.2 | 0.05 |
| Page, 2013 | 16% (12%, 19%) | 255 | <0.001 | 87.5 | 0.06 |
| Gopal, 2014 | 16% (13%, 19%) | 254.9 | <0.001 | 87.5 | 0.06 |
| Sameen, 2014 | 16% (13%, 20%) | 238.4 | <0.001 | 86.6 | 0.05 |
| Ahmed, 2015 | 15% (12%, 19%) | 241.9 | <0.001 | 86.8 | 0.06 |
| Anjum, 2016 | 16% (13%, 19%) | 249.1 | <0.001 | 87.1 | 0.05 |
| Thuo, 2017 | 16% (13%, 19%) | 208.3 | <0.001 | 84.6 | 0.05 |
| **Overall** | **16% (13%, 19%)** | **255.5** | **<0.001** | **87.1%** | **0.05** |

Supplementary Table 4: Leave-one-out sensitivity analyses of random-effects association between malnutrition and urinary tract infection in children

| Study omitted | OR (95% CI) | P-value (OR) | Q-statistic for heterogeneity | P-value (Q) | Proportion of variation attributable to heterogeneity (I^2^; %) | Between-study variance (Tau^2^) |
| --- | --- | --- | --- | --- | --- | --- |
| Buchanan, 1971 | 3.26 (1.63, 6.50) | 001 | 12.3 | 0.06 | 51.1 | 0.40 |
| Freyre 1973 | 3.22 (1.46, 7.06) | 004 | 13.8 | 0.03 | 56.6 | 0.56 |
| Bodaghi, 1978 | 2.36 (1.15, 4.87) | 020 | 11.4 | 0.08 | 47.2 | 0.41 |
| Banapurmath, 1994 | 2.64 (1.30, 5.36) | 007 | 14.2 | 0.03 | 57.8 | 0.49 |
| Jeena, 1995 | 3.14 (1.34, 7.35) | 008 | 14.5 | 0.02 | 58.6 | 0.69 |
| Caksen, 2001 | 3.25 (1.48, 7.11) | 003 | 13.5 | 0.04 | 55.6 | 0.55 |
| Bagga, 2003 | 2.34 (1.19, 4.62) | 014 | 11.7 | 0.07 | 48.5 | 0.37 |
| Gopal, 2014 | 2.49 (1.28, 4.82) | 007 | 12.4 | 0.05 | 51.6 | 0.38 |
| **Overall** | **2.80 (1.41, 5.54)** | **003** | **15.1** | **0.04** | **53.6** | 0.**47** |
